# Uptake of the Interim Canada Dental Benefit: an investigation of data from the first 18 months of the program

**DOI:** 10.3389/froh.2024.1481423

**Published:** 2024-10-22

**Authors:** Saif Goubran, Vivianne Cruz de Jesus, Anil Menon, Olubukola O. Olatosi, Robert J. Schroth

**Affiliations:** ^1^ Dr. Gerald Niznick College of Dentistry, Rady Faculty of Health Sciences, University of Manitoba, Winnipeg, MB, Canada; ^2^ Children’s Hospital Research Institute of Manitoba, Winnipeg, MB, Canada; ^3^ Shared Health Manitoba, Winnipeg, MB, Canada

**Keywords:** insurance, dental, child, health policy, public health dentistry, access to care

## Abstract

**Introduction:**

In 2022, the Government of Canada introduced the Interim Canada Dental Benefit (CDB) to support Canadian families with children <12 years of age. This program operated from October 1, 2022, to June 30, 2024, with two application periods. The purpose of this study was to analyze data on applications accepted by the Canada Revenue Agency (CRA) during the first 18 months of the program.

**Methods:**

This study used available data sourced from the CRA for applicants as of March 29, 2024, and assessed as of April 5, 2024. Data covered the entirety of the first period (October 1, 2022–June 30, 2023) of the Interim CDB and the first nine months of the second period (July 1, 2023–March 29, 2024). The rate of child participation was calculated using population data from Statistics Canada (2021).

**Results:**

Over the first 18 months of the Interim CDB, a total of 410,920 applications were submitted and $403M distributed; $197M for 204,270 applications in period 1 and $175M for 173,160 applications in the first nine months of period 2. Overall, 321,000 children received the Interim CDB in period 1 and 282,130 children received the Interim CDB in the first nine months of period 2. A total of 91.8% of applicants had a net family income <$70,000, receiving the maximum benefit amount. The provinces with the highest rate of child participation were Manitoba (77.1/1,000 period 1; 74.9/1,000 period 2), Ontario (82.5/1,000 period 1; 72.2/1,000 period 2), Nova Scotia (73.4/1,000 period 1; 71.1/1,000 period 2), and Saskatchewan (72.3/1,000 period 1; 68.2/1,000 period 2). Overall, projections suggest that there will be an increase in the number of applications approved in period 2 compared to period 1.

**Conclusions:**

Uptake in the first three quarters of period 2 remained consistent and in many instances, revealed higher rates of applications by parents for the Interim CDB. However, it is uncertain how much of the funds were directly used for dental care. Analyzing this data will aid in policy recommendation for enhancement of the Canadian Dental Care Program.

## 1 Introduction

While Canada prides itself on its universal health care system, the oral health care needs of many Canadians have seemingly fallen through the cracks. The 2007–2009 Canadian Health Measures Survey revealed that nearly one-third of Canadians lack dental insurance (1). Lower-income families and those without insurance are three to four times more likely to not obtain dental care than higher-income Canadians, with 16.5% declining recommended care due to cost. Furthermore, 56.8% of Canadian children ages 6–11 are affected by dental caries. However, no national data exists on the prevalence of dental caries among children <6 years of age. Additionally, many Canadian children experience significant oral health disparities, particularly those from low-income households, Indigenous communities, newcomer populations, and those dwelling in rural and remote parts of the country (2, 3). Rates of surgery to treat early childhood caries are high and are significantly associated with low socioeconomic status (3).

Children most at risk of developing caries are those facing barriers to accessing oral health care systems (2). Barriers to accessing care are disproportionately faced by First Nation, Inuit, and Métis children, those living in remote communities, recent refugees and immigrants, and those of low socioeconomic status (4). Children suffering from severe caries face a reduced quality of life due to pain, disturbed sleep, altered growth patterns, low confidence, missed school days, and malnutrition (5). The extent of caries may also warrant dental surgery under general anesthesia, which can be associated with potential complications for young children (6).

In March of 2022, the Canadian government made a historic announcement about its intent to introduce a national dental insurance program for at-risk Canadians lacking dental insurance, in response to political pressure from another political party that it had entered a governing coalition with through a confidence and supply agreement. As part of the announcement, the government pledged $5.3B to fund low-income families to cover dental expenses. Since the development and implementation of a national dental insurance program for children, seniors, and those with special healthcare needs would take up to two years, the federal government implemented the Interim Canada Dental Benefit (CDB) for children <12 years of age as a stop-gap measure. The Interim CDB was intended to be a precursor to the Canadian Dental Care Plan (CDCP). The Interim CDB was introduced as a direct benefit payment to parents to support dental expenditures for their children.

Only families with annual incomes <$90,000 were eligible to receive the CDB for children <12 years of age (7–9). Families with private insurance or insurance from an employer-sponsored plan were ineligible to receive the benefit. Applicants must have filed their income tax for the previous year and received the Canada Child Benefit (i.e., a tax-free monthly payment made to eligible families to help with the cost of raising children under 18 years of age). However, applicants receiving government dental benefits such as the Non-Insured Health Benefits (for registered First Nations and Inuit Peoples) or Employment and Income Assistance (i.e., social assistance) were also eligible to receive the interim CDB if their dental care expenses for a child were not fully reimbursed. Families with an annual income between $80,000 and $89,999 were eligible to receive $260 for each eligible child. Those with an annual income between $70,000 and $79,999 received $390 for each eligible child. Finally, families with an annual income under $70,000 received $650 for each eligible child. Funds were distributed by the Canada Revenue Agency (CRA). Parents accessing the Interim CDB received up to a maximum of two payments of up to $650 over the two periods of the program (10–12). The Regular Period 1 of the Interim CDB included eligible children who received dental treatment between October 1, 2022, and June 30, 2023. The Regular Period 2 of the CDB included eligible children who received dental treatment between July 1, 2023, and June 30, 2024.

As the Interim CDB sunset on June 30, 2024, applications for the CDCP commenced on June 27, 2024, providing coverage to a greater range of uninsured Canadians with a family income <$90,000 (7, 13). A national program of such scale presents the opportunity for evaluation and ongoing policy development.

The purpose of this study was to analyze data collected by the Government of Canada on applications made and accepted by the CRA during the entirety of the first period and the first nine months of the second period of the Interim CDB. This study uses available aggregated data from the first 18 months of the program sourced from the CRA for applicants as of March 29, 2024, and assessed as of April 5, 2024.

## 2 Methods

This study analyzed data supplied by the CRA during the first 18 months of the Interim CDB, up to March 29, 2024, and assessed as of April 5, 2024. Public aggregated data was accessed from the Government of Canada Open Data Portal: https://open.canada.ca/data/en/dataset/69035265-2714-4ffa-af3f-fa850209b616 (14, 15). Ethics approval was not required for this quantitative study as it involved exclusively de-identified aggregated data publicly accessible from the government of Canada. The Regular Period 1 data include data on applicants that had applied, received the Interim CDB, and sought dental care for their child(ren) between October 1, 2022, to June 30, 2023 (16). The Regular Period 2 data include applicants that had dental treatment, applied, received the benefit, and sought dental care for their child(ren) between July 1, 2023, to June 30, 2024 (16).

The Additional Period 1 data include applicants whose children had dental treatment with costs that exceeded $650 between October 1, 2022, and June 30, 2023 (16). These applicants were eligible to apply and receive an additional $650 within the second pay period, granted they could not apply for the Regular Period 2 of the CDB (16). The Additional Period 2 data include applicants who had children whose cost for dental treatment exceeded $650 between July 1, 2023, and June 30, 2024 (16). These applicants were eligible to apply and receive an additional $650 within the second period, granted they had not applied within the Regular Period 1 of the CDB (16).

Variables of interest included the number of applications submitted to the CRA, the number of unique applicants, the gender of the approved applicants, the age of the approved applicants, the number of children receiving the benefit, and the total amount of funding received. Such variables were presented as distributed by provinces and territories, age grouping of children, and family net income.

The number of applications and the applicant's province/territory of residence were obtained from the Canada Dental Benefit file. A unique applicant was an individual. An applicant may have applied for more than one child. The residence of the applicant was as of the date of receiving the application. All amounts were rounded to the nearest thousand and were in thousands of dollars, and all counts were rounded to the nearest ten (14, 15). Due to rounding, suppression, and/or double counting for shared custody, the sum of the data may not add to the total (14, 15). Information that has been suppressed for confidentiality purposes was indicated by a “0” (14, 15). Suppressed information also included valid zeros (14, 15). Individuals whose gender is non-binary were represented in the gender-diverse category (14, 15).

Rates of children with the Interim CDB per 1,000 were calculated by dividing the number of children with the benefit by the number of Canadians aged 0–11, by province or territory, based on census 2021 data available from Statistics Canada (17). Analysis included descriptive statistics (frequencies and proportions) were done in Microsoft Excel.

## 3 Results

**Figure 1:**
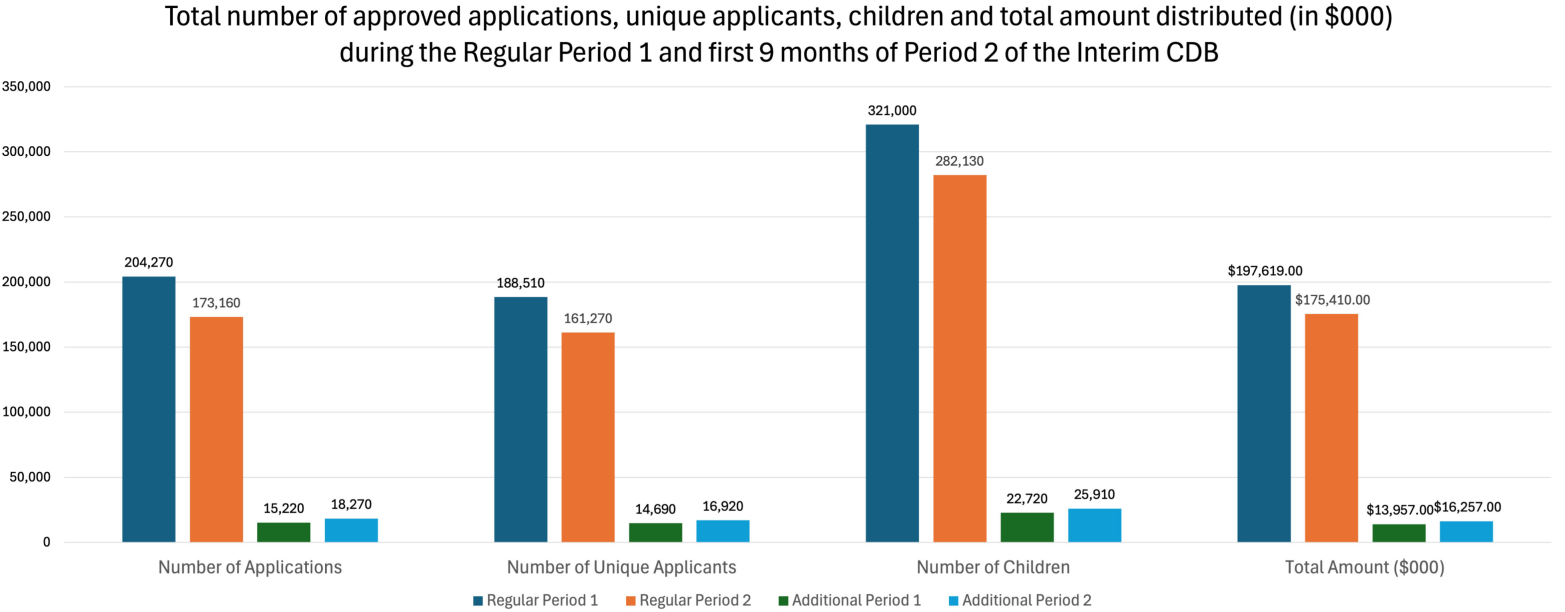
Total number of approved applications, unique applicants, children, and total amount distributed (in $000) during the regular period 1^a^, first 9 months of regular period 2^b^, additional period 1^c^, and first 9 months of additional period 2^d^. ^a^For services received between October 1, 2022, to June 30, 2023. ^b^For services received between July 1, 2023, to March 29, 2024. ^c^For applicants who paid more than $650 for their child's dental services during the Regular Period 1 as of March 29, 2024. ^d^For applicants who paid more than $650 for their child's dental services during the Regular Period 2 as of March 29, 2024.

The number of applications to the Interim CDB approved by the CRA, the number of unique applicants, and the total amount of funds distributed are reported in Table 1 and Figure 1. During the first 18 months of the Interim CDB, 410,920 applications were submitted (including both regular and additional periods), and $403M was distributed. During the Regular Period 1 (October 1, 2022, to June 30, 2023), 204,270 applications made by 188,510 unique applicants, resulting in 321,000 children <12 years of age approved to receive the Interim CDB (Tables 1, 2). A total of $197M was distributed by the CRA. During the first nine months of Regular Period 2 (July 1, 2023, to March 29, 2024), there were 173,160 approved applications made by 161,270 unique applicants resulting in 282,130 children <12 years of age receiving the Interim CDB (Tables 1, 2). The CRA distributed a total of $175M during the first nine months of Regular Period 2.

**Table 1:** Number of approved interim CDB applications, unique applicants, and total amount distributed (in $000) by province/territory during regular period 1^a^, first 9 months of regular period 2^b^, additional period 1^c^, and first 9 months of additional period 2^d^.

	Number of applications				Number of unique applicants				Total amount ($000)			
P/T	Regular period 1	Regular period 2	Additional period 1	Additional period 2	Regular period 1	Regular period 2	Additional period 1	Additional period 2	Regular period 1	Regular period 2	Additional period 1	Additional period 2
Newfoundland and Labrador	1,900	1,700	170	210	1,720	1,590	160	200	1,758	1,674	143	197
Prince Edward Island	520	490	40	50	470	450	40	50	470	433	39	44
Nova Scotia	5,070	4,720	470	610	4,590	4,330	460	570	4,989	4,833	430	519
New Brunswick	3,250	3,170	290	420	2,920	2,880	270	390	3,144	3,146	253	362
Quebec	37,830	30,380	1,710	2,330	34,250	27,550	1,640	2,120	33,639	28,538	1,365	1,880
Ontario	90,940	77,170	7,130	7,770	85,030	72,830	6,920	7,280	90,695	80,069	6,544	6,873
Manitoba	9,210	8,650	830	1,300	8,100	7,760	780	1,160	9,775	9,549	937	1,299
Saskatchewan	7,800	6,990	960	1,280	6,800	6,290	900	1,150	8,123	7,675	1,033	1,275
Alberta	24,140	20,250	2,060	2,480	22,340	19,010	1,990	2,300	23,862	21,107	1,931	2,287
British Columbia	23,190	19,270	1,470	1,720	21,910	18,260	1,430	1,610	20,712	17,970	1,186	1,415
Yukon	60	50	0	10	60	50	0	10	51	43	0	7
Northwest Territories	230	190	0	30	200	170	0	30	256	214	0	32
Nunavut	130	140	50	70	110	120	50	50	133	156	47	66
Total	204,270	173,160	15,220	18,270	188,510	161,270	14,690	16,920	197,619	175,410	13,957	16,257

^a^ For services received between October 1, 2022, and June 30, 2023.

^b^ For services received between July 1, 2023, and March 29, 2024.

^c^ For applicants who paid more than $650 for their child's dental services during the Regular Period 1 as of March 29, 2024.

^d^ For applicants who paid more than $650 for their child's dental services during the Regular Period 2 as of March 29, 2024.

**Table 2:** Number of approved interim CBD children and rate of child participation by province/territory during the regular period 1^a^, first 9 months of regular period 2^b^, additional period 1^c^, and first 9 months of additional period 2^d^.

	Number of children				Rate of child participation (per 1,000)			
P/T	Regular period 1	Regular period 2	Additional period 1	Additional period 2	Regular period 1	Regular period 2	Additional period 1	Additional period 2
Newfoundland and Labrador	2,790	2,660	230	310	53.3	50.8	4.4	5.9
Prince Edward Island	780	730	70	80	42.6	39.9	3.8	4.4
Nova Scotia	7,880	7,630	680	820	73.4	71.1	6.3	7.6
New Brunswick	5,050	5,040	410	580	58.3	58.1	4.7	6.7
Quebec	57,400	47,930	2,360	3,100	52.2	43.6	2.1	2.8
Ontario	145,610	127,410	10,610	10,900	82.5	72.2	6.0	6.2
Manitoba	15,530	15,080	1,490	2,020	77.1	74.9	7.4	10.0
Saskatchewan	12,810	12,090	1,620	1,990	72.3	68.2	9.1	11.2
Alberta	39,120	34,230	3,170	3,670	60.9	53.3	4.9	5.7
British Columbia	34,230	29,350	1,970	2,290	60.9	52.2	3.5	4.1
Yukon	90	70	0	10	16.5	12.8	0.0	1.8
Northwest Territories	410	340	0	50	59.9	49.7	0.0	7.3
Nunavut	220	240	80	100	22.1	24.1	8.0	10.1
Total	321,000	282,130	22,720	25,910	67.8	59.6	4.8	5.5

^a^ For services received between October 1, 2022, and June 30, 2023.

^b^ For services received between July 1, 2023, and March 29, 2024.

^c^ For applicants who paid more than $650 for their child's dental services during the Regular Period 1.

^d^ For applicants who paid more than $650 for their child's dental services during the Regular Period 2.

During Additional Period 1, 15,220 supplemental applications made by 14,690 unique applicants for 22,720 children were approved (Tables 1, 2). A total of $14M was distributed by the CRA. As of March 29, 2024, 18,270 supplemental applications were approved as part of Additional Period 2, made by 16,920 unique applicants for 25,910 children, with the CRA distributing over $16M.

As displayed in Table 1, there were a greater number of applicants to the Interim CDB during the Regular Period 1 when compared to the first nine months of Regular Period 2; however, there were a greater number of applicants during the first nine months of the Additional Period 2 of the program when compared to the Additional Period 1. Families in Ontario received the greatest proportion of Interim CDB funding in both periods. During the first nine months of the second period of the Interim CDB, over $80M went to Ontario residents (45.6%), while $28.5M went to families in Quebec, $21.1M to Alberta, and over $17.9M to British Columbia. Families in the Yukon, Nunavut, and Northwest Territories received the least funding during the second benefit period, totaling $0.413M (0.24%).

Table 2 reveals that the greatest number of children who received the Interim CDB during Period 1 and Period 2 were from Ontario, followed by Quebec, Alberta and British Columbia. The territories had the fewest numbers of children who received the Interim CDB during Period 1 and Period 2. However, taking into consideration population differences between provinces and territories, the rate of child participation per 1,000 children was calculated using population data from Statistics Canada (2021) for children ages 0–11 and displayed in Table 2 (18). The national rate of child participation was 59.6/1,000 during the first nine months of the Regular Period 2 compared to 67.8/1,000 for the Regular Period 1 (12). The provinces with the highest rate of child participation were Manitoba (77.1/1,000 period 1; 74.9/1,000 period 2), Ontario (82.5/1,000 period 1; 72.2/1,000 period 2), Nova Scotia (73.4/1,000 period 1; 71.1/1,000 period 2), and Saskatchewan (72.3/1,000 period 1; 68.2/1,000 period 2), all having rates of child participation greater than the national rates. Rates of unique applicants to the first nine months of the additional period funding streams (when dental costs exceeded $650) were 4.8/1,000 and 5.5/1,000 for the Additional Period 1 and Additional Period 2, respectively.

As displayed in Table 3, during the Regular Period 1, 45.6% of unique applicants had a net adjusted family income < $30,000, compared to 54.6% of unique applicants during the first nine months of the Regular Period 2. Over the 18 months of the Interim CDB, 91.8% of applicants had a net family income < $70,000. Overall, 90.8% of children received the maximum benefit amount (i.e., $650) during Period 1 of the program, compared to 92.6% during the first nine months of the second period of the Interim CDB. The proportion of children whose applications for additional funding were approved and received $650 were 92% during Additional Period 1% and 95% during Additional Period 2.

**Table 3:** Number of approved interim CDB applications, unique applicants, children, and total amount (in $000) by adjusted net family income during regular period 1^a^, first 9 months of regular period 2^b^, additional period 1^c^, and first 9 months of additional period 2^d^.

		Number of unique applicants				Number of children			
Family net income	Payment per child	Regular period 1	Regular period 2	Additional period 1	Additional period 2	Regular period 1	Regular period 2	Additional period 1	Additional period 2
Less than $10,000	$650	19,370 (10.3%)	24,410 (15.1%)	1,640 (11.2%)	3,510 (20.7%)	34,670 (10.8%)	44,790 (15.9%)	2,700 (11.9%)	5,750 (22.2%)
$10,000–$19,999	$650	29,060 (15.4%)	34,900 (21.6%)	2,640 (18.0%)	4,280 (25.3%)	51,650 (16.1%)	63,020 (22.3%)	4,320 (19.0%)	6,610 (25.5%)
$20,000–$29,999	$650	37,500 (19.9%)	28,840 (17.9%)	3,260 (22.2%)	2,940 (17.4%)	64,130 (20.0%)	51,110 (18.1%)	4,960 (21.8%)	4,490 (17.3%)
$30,000–$39,999	$650	30,610 (16.2%)	22,640 (14.0%)	2,340 (15.9%)	2,140 (12.6%)	51,710 (16.1%)	38,900 (13.8%)	3,540 (15.6%)	3,210 (12.4%)
$40,000–$49,999	$650	23,360 (12.4%)	16,540 (10.3%)	1,690 (11.5%)	1,380 (8.2%)	39,200 (12.2%)	28,040 (9.9%)	2,520 (11.1%)	1,990 (7.7%)
$50,000–$59,999	$650	17,710 (9.4%)	12,460 (7.7%)	1,120 (7.6%)	1,070 (6.3%)	29,580 (9.2%)	20,840 (7.4%)	1,650 (7.3%)	1,570 (6.1%)
$60,000–$69,999	$650	13,480 (7.2%)	9,630 (6.0%)	810 (5.5%)	760 (4.5%)	22,490 (7.0%)	16,340 (5.8%)	1,180 (5.2%)	1,120 (4.3%)
$70,000–$79,999	$390	10,320 (5.5%)	7,080 (4.4%)	670 (4.6%)	520 (3.1%)	16,960 (5.3%)	11,780 (4.2%)	1,020 (4.5%)	730 (2.8%)
$80,000–$89,999	$260	7,110 (3.8%)	4,780 (3.0%)	540 (3.7%)	330 (2.0%)	11,550 (3.6%)	7,990 (2.8%)	840 (3.7%)	480 (1.9%)
Total		188,510	161,270	14,690	16,920	321,000	282,130	22,720	25,910

^a^ For services received between October 1, 2022, and June 30, 2023.

^b^ For services received between July 1, 2023, and March 29, 2024.

^c^ For applicants who paid more than $650 for their child's dental services during the Regular Period 1 as of March 29, 2024.

^d^ For applicants who paid more than $650 for their child's dental services during the Regular Period 2 as of March 29, 2024.

Table 4 displays the ages of accepted applicants to the Interim CDB. During Regular Period 1, 83.7% of the applicants were between the ages of 25 and 44, compared to 85% during the first nine months of the second period. Similarly, during the first nine months of Additional funding streams, 85% of the applicants were between 25 and 44 (data not shown). The same pattern was true for all provinces and territories.

**Table 4:** Number of approved interim CDB unique applicants by province and territory, and age group during the regular period 1^a^ and first 9 months of regular period 2^b^.

Age group *N* (%)														
	<25		25–34		35–44		45–54		55–64		65+		Total^c^	
P/T	RP1	RP2	RP1	RP2	RP1	RP2	RP1	RP2	RP1	RP2	RP1	RP2	RP1	RP2
Newfoundland and Labrador	110 (6.4)	80 (5.0)	790 (45.9)	790 (49.7)	680 (39.5)	590 (37.1)	120 (7.0)	110 (6.9)	20 (1.2)	10 (0.6)	0 (0.0)	0 (0.0)	1,720	1,590
Prince Edward Island	10 (2.1)	20 (4.4)	210 (44.7)	200 (44.4)	200 (42.6)	180 (40.0)	60 (12.8)	50 (11.1)	0 (0.0)	0 (0.0)	0 (0.0)	0 (0.0)	470	450
Nova Scotia	250 (5.5)	280 (6.5)	2,220 (48.4)	2,210 (51.0)	1,720 (37.5)	1,500 (34.6)	340 (7.4)	300 (6.9)	40 (0.9)	30 (0.7)	20 (0.4)	10 (0.2)	4,590	4,330
New Brunswick	140 (4.8)	170 (5.9)	1,410 (48.3)	1,390 (48.3)	1,110 (38.0)	1,070 (37.2)	230 (7.9)	220 (7.6)	10 (0.3)	20 (0.7)	0 (0.0)	0 (0.0)	2,920	2,880
Quebec	590 (1.7)	620 (2.3)	11,090 (32.4)	9,940 (36.1)	17,170 (50.1)	13,270 (48.2)	5,090 (14.9)	3,490 (12.7)	250 (0.7)	190 (0.7)	50 (0.2)	30 (0.1)	34,250	27,550
Ontario	2,300 (2.7)	2,440 (3.4)	31,100 (36.6)	28,540 (39.2)	40,280 (47.4)	33,510 (46.0)	10,370 (12.2)	7,630 (10.5)	710 (0.8)	500 (0.7)	180 (0.2)	130 (0.2)	85,030	72,830
Manitoba	440 (5.4)	500 (6.4)	3,460 (42.7)	3,430 (44.2)	3,410 (42.1)	3,150 (40.6)	680 (8.4)	570 (7.3)	80 (1.0)	100 (1.3)	20 (0.3)	20 (0.3)	8,100	7,760
Saskatchewan	550 (1.7)	550 (8.7)	3,040 (44.7)	2,920 (46.4)	2,580 (37.9)	2,280 (36.2)	510 (7.5)	450 (7.2)	90 (1.3)	80 (1.3)	20 (0.3)	10 (0.2)	6,800	6,290
Alberta	720 (3.2)	770 (4.1)	8,660 (38.8)	7,910 (41.6)	10,480 (46.9)	8,540 (44.9)	2,230 (10.0)	1,610 (8.5)	150 (0.7)	120 (0.6)	50 (0.2)	30 (0.2)	22,340	19,010
British Columbia	410 (1.9)	410 (2.2)	6,650 (30.4)	6,180 (33.8)	11,270 (51.4)	9,160 (50.2)	3,300 (15.1)	2,320 (12.7)	190 (0.9)	140 (0.8)	80 (0.4)	40 (0.2)	21,910	18,260
Yukon	0 (0.0)	0 (0.0)	20 (33.3)	20 (40.0)	30 (50.0)	30 (60.0)	0 (0.0)	0 (0.0)	0 (0.0)	0 (0.0)	0 (0.0)	0 (0.0)	60	50
Northwest Territories	10 (5.0)	0 (0.0)	120 (60)	90 (52.9)	60 (30.0)	50 (29.4)	0 (0.0)	0 (0.0)	0 (0.0)	0 (0.0)	0 (0.0)	0 (0.0)	200	170
Nunavut	20 (18.2)	10 (8.3)	60 (54.6)	70 (58.3)	30 (27.3)	30 (25.0)	0 (0.0)	0 (0.0)	0 (0.0)	0 (0.0)	0 (0.0)	0 (0.0)	110	120
Total	5,550 (2.9)	5,860 (3.6)	68,830 (36.5)	63,680 (39.5)	89,010 (47.2)	73,340 (45.5)	22,950 (12.2)	16,770 (10.4)	1,550 (0.8)	1,180 (0.7)	440 (0.2)	280 (0.2)	188,510	161,270

^a^ For services received between October 1, 2022, and June 30, 2023.

^b^ For services received between July 1, 2023, and March 29, 2024.

^c^ Includes individual applicants with unknown age.

Based on the aggregate data provided, a majority of individuals filing applications to the CRA during the 18 months of the program were female. Over the duration of the program, 362,240 of the 381,390 applicants, or 95.0%, were female. When considering the distribution of funds, $386,699,000 were distributed to female beneficiaries and $15,873,000 to males. Only 36 applicants identified as gender diverse.

**Figure 2:**
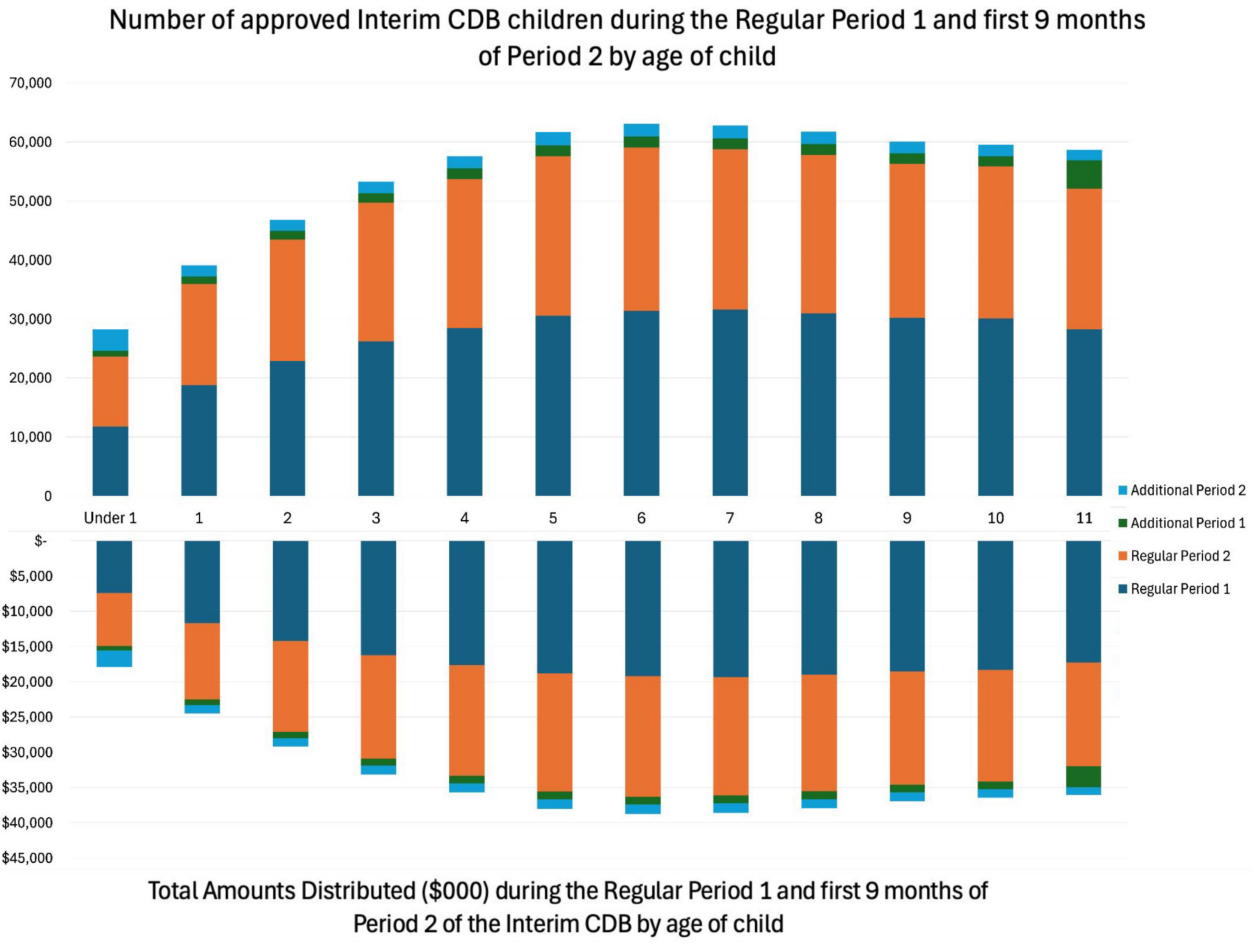
Number of approved interim CDB children and the total approved amount by age regular period 1^a^, first 9 months of regular period 2^b^, additional period 1^c^, and first 9 months of additional period 2^d^. ^a^For services received between October 1, 2022, to June 30, 2023. ^b^For services received between July 1, 2023, to March 29, 2024. ^c^For applicants who paid more than $650 for their child's dental services during the Regular Period 1 as of March 29, 2024. ^d^For applicants who paid more than $650 for their child's dental services during the Regular Period 2 as of March 29, 2024.

Figure 2 reports on the number of children receiving the Interim CDB benefit and the total amounts distributed by age during Period 1, the first nine months of Period 2, and the Additional Periods. As displayed in Figure 2, infants and preschool children accounted for the smallest proportion of recipients of the Interim CDB. During the first nine months of the Regular Period 2, 54.1% of the recipients were less than 6 years of age, receiving $112M. The number of children receiving the benefit and amount received increased with age up to age 4 years; this distribution tended to stabilize after the age of 4 years. The highest amount of funding was received by the 7-year-old age group ($19.5M). 11-year-old children received the most Additional Period 1 funding ($4.78M).

Table 5 displays projected totals for the number of applications, number of unique applicants, number of children, and total amount distributed ($000) by June 30, 2024. Projections for the Regular Period 2, Additional Period 1, and Additional Period 2 were calculated by taking the data from the first nine months of the second period (up to March 29, 2024) and projecting it over 12 months (up to June 30, 2024). Using these projections, over 230,880 applications are estimated to be approved between July 1, 2023, and June 30, 2024, made by over 215,027 unique applicants for over 376,173 children <12 years of age. It is projected that the CRA will distribute a total of over $233M.

**Table 5:** Total number of approved interim CDB applications, unique applicants, number of children, and total amount distributed (in $000) during the regular period 1^a^, and projected totals for regular period 2^b^, additional period 1^c^, and additional period 2^d^ by June 30, 2024.

	Number of applications	Number of unique applicants	Number of children	Total amount ($000)
Regular period 1	204,270	188,510	321,000	197,619
Projected regular period 2	230,880	215,027	376,173	233,880
Projected additional period 1	20,293	19,587	30,293	18,609
Projected additional period 2	24,360	22,560	34,547	21,676
Projected totals	547,893	508,520	869,013	537,657

^a^ For services received between October 1, 2022, and June 30, 2023.

^b^ Projection for services received between July 1, 2023, and June 30, 2024.

^c^ Projection for applicants who paid more than $650 for their child's dental services during the Regular Period 1 by June 30, 2024.

^d^ Projection for applicants who paid more than $650 for their child's dental services during the Regular Period 2 by June 30, 2024.

## 4 Discussion

This study reviewed aggregated data from the first 18 months of the Interim CDB program, a time-limited financial benefit provided directly to families with children <12 years of age, from families with household incomes < $90.000. Overall, our study showed a progressive increase in the number of applications and eligible children benefiting from the Interim CBD program by the end of the program on June 30, 2024 (12). Rates of applications to the CRA for the Interim CDB also remained consistent. These findings suggest that there is a proportion of families with financial barriers to accessing oral health care for their children <12 years of age. The 2007–2009 Canadian Health Measures Survey revealed that nearly one-third of Canadians lack dental insurance (1). Families with no insurance are four times more likely to not obtain dental care compared to higher-income Canadians, with 16.5% declining recommended care due to cost (1). The Canadian Academy of Health Sciences reports that 80% of high-income families have dental insurance, while 50% of low-income families lack dental insurance (4). Those with the fewest barriers to oral health care receive the most support, while those with the greatest need are forced to pay out-of-pocket. A 2020 Ontario study suggests that access to dental insurance increases the proportion of children visiting the dentist (19). Furthermore, the study suggests that access to dental care more significantly impacts the lowest-income groups when compared to the highest-income groups (19). This present study on the Interim CDB corroborates with the findings from this study, showing that children from the lowest-income bracket had the highest uptake of the Interim CDB.

More funds were distributed during the nine months of Regular Period 1 than during the first nine months of Regular Period 2 ($198M compared to $175M). This may be attributed to a lack of awareness that parents were eligible to apply for a second year of the interim CDB for their children, less advertising during Period 2, and even potential dissatisfaction or negative experiences with the program during Period 1. Dental providers may also have been unaware and failed to encourage parents to reapply for the second funding period. It could also be that the growing awareness and media attention regarding the new CDCP launching for children <18 years of age on July 1, 2024, also might have overshadowed Period 2 of the interim CDB. However, when forecasting the full 12 months of Regular Period 2 based on the first nine months, it is estimated that over $233M will be distributed to Canadian families by June 30, 2024. It will be interesting to see if these projections match the final quarter of Period 2 once those data are available from CRA, as families may have misunderstood growing national dentist opposition to the upcoming CDCP as opposition to the Interim CDB.

Furthermore, the decrease in Regular Period 2 applicants can be a result of children's dental work exceeding $650, leading families to need to apply for supplemental pay periods during the second period of the benefit as opposed to the Regular Period 2 of the program. Additional Period 1 applicants included those with dental treatment costing more than $650 during the first period of the benefit, making them eligible to receive an additional $650 within the second pay period, granted they cannot apply for the Regular Period 2 of the Interim CDB. This was the circumstance for 7.0% of children who applied and received insufficient funding to cover their dental costs during the first period of the benefit. As 11-year-old children received the most Additional Period 1 funding, this age group may be experiencing unmet needs more greatly than other age groups, leading to the need for supplemental funding when costs exceeded $650. Additional Period 2 applicants included those with dental treatments costing more than $650 during the second period of the benefit, making them eligible to receive an additional $650 within the second period, granted they had not applied within the Regular Period 1 of the Interim CDB. Such was the case for 9.2% of children who received the benefit during the second period of the benefit. These individuals may include families that did not apply to the program during the first period. Reasons parents may not have applied for the benefit during the first period can include a lack of awareness due to insufficient advertisement, difficulty understanding the objective of the program due to language barriers, or lack of access to a dental care provider during the time of the first period. Nevertheless, $650 from one period of the benefit was likely inadequate to cover the dental expenses for the child (12). It has recently been reported that in most Canadian provinces, $650 would likely be able to cover the cost associated with an examination, radiographs, cleaning, fluoride varnish application, and other preventative sealants but likely would not be sufficient to cover the cost of a restorative procedure (12). With children from low-income families being more at risk for caries, $650 may be insufficient to cover the needs of many children (5). Overall, the relatively low proportion of supplementation applications may be due to limited awareness among parents and dental providers of this mechanism to access additional funds to cover their child's dental expenses.

Interestingly, while Regular Period 1 had a greater number of applicants, Regular Period 2 had a greater proportion of applicants with an adjusted family income < $30,000 (45.6% in Period 1 as compared to 54.6% in first nine months of period 2). This could result from an overall decrease in applicants for Regular Period 2 but more awareness of the program amongst low-income families. The increased awareness among low-income families may be attributed to parents being encouraged to apply for the program by community clinics and agencies or through word of mouth within the low-income community. Schroth et al. 2024 report that higher-income groups may lack awareness of the benefit, find the program unappealing due to the reduced benefit, or have less need for government support (12) However, the fact that many applications were made by parents from the <$70,000 income bracket means that most children received the full $650 payment. This is important as it means that this uptake by lower income families will influence the costing of the new CDCP and discussions around oral health equity and possible copayment scenarios. It is also interesting that the majority of applicants were female, suggesting that the responsibility for children's oral health care and health care in general lies with women. While this may not be entirely surprising it should inform how the CDCP is promoted to families moving forward.

In terms of provincial differences between the number of applications accepted and the amount of funding distributed, the same outcomes are observed between the first and second periods of the benefit (12). The provinces with the highest rate of child participation were Manitoba, Ontario, Nova Scotia, and Saskatchewan. Ontario had the highest participation during Regular Period 1, and Manitoba had the highest participation during Regular Period 2. Results are congruent with reports from Menon et al. 2024, indicating that Manitoba and Saskatchewan may have higher than national rates of participation in the program due to reported poorer access to dental services as compared to other provinces (20). Significantly more parents of children <12 years of age from these two prairie provinces reported access to oral health care challenges than from any other part of the country (20). The pediatric oral health access to care issues in Manitoba and Saskatchewan may be explained by past reversals of social dental programs in these provinces. Furthermore, the national average for the rate of child participation per 1,000 children during Additional Period 1 and Additional Period 2 was 4.8 and 5.5, respectively. Both Manitoba (7.4 and 10.0) and Saskatchewan (9.1 and 11.2) had rates above the national average. The need for additional payments indicates that the treatment of children in these provinces exceeded the $650 distributed, emphasizing the unmet treatment needs of children in these provinces.

The territories displayed lower child participation during the first 18 months of the benefit. Such a result may be related to the access to care challenges northern communities face. When applying for the Interim CDB, applicants must provide the location of a dental practice they will attend for a scheduled appointment and provide the information of a dental practitioner (12). This requirement may be challenging for individuals living in remote or underserved areas. Given the high proportion of First Nations and Inuit people living in the territories, many children may have federal dental benefits from the Non-Insured Health Benefits (NIHB) Program and may not have seen any benefit in additionally applying for the interim CDB. However, it remains unclear whether there is any advantage for First Nations and Inuit people with NIHB Program dental benefits to apply for the CDCP.

Canadians of all ages, particularly children, would benefit from a government-funded program to subsidize the cost of dental care, and the Interim CDB appears to be a great step forward. Figure 2 displays the age groupings of children receiving the Interim CDB. It may come as a welcome surprise that children <1 year of age accounted for 10.3% of children receiving the benefit, as engagement of children this age with the benefit was anticipated to be minimal. Low engagement with this age was anticipated due to a lack of confidence in providers to work with young children as well as a lack of parental awareness of the importance of oral health care for young children. Findings from this study may be a result of dental offices encouraging parents to apply for the benefit and parents taking ownership to follow through with the application. While the engagement with younger children is impressive, there is still work to be done. Educating parents and oral health care providers on the importance of a first visit prior to the child's first birthday and the establishment of a dental home can prove to decrease the progression of ECC within communities (21).

This study is not without limitations. As the study used aggregate data, direct comparisons between the two periods of the Interim CDB cannot be made. For instance, since the data provided by CRA are aggregated, it is unclear if the applicants in Period 1 reapplied for the Interim CDB in Period 2, making It challenging to determine whether individuals are new or repeat applicants. Additionally, there was no direct information available on specific regions within Canadian provinces and territories preventing us from investigating whether the uptake of the Interim CDB was higher in areas with greater oral health needs. Fortunately, aggregated data on household income were available, allowing for an assessment of applications from the poorest families in Canada (i.e., Table 3 household income <$30,000), which serves as proxy measure for socioeconomics and possibly healthcare access and health status.

Beyond financial barriers, several other limitations may have affected the program's reach and effectiveness. Geographic barriers, particularly in rural and remote areas, likely limited access to dental care despite financial assistance. These areas often face shortages of dental providers, making it difficult for families to access services even when they qualify for the benefit. Similarly, cultural and language barriers may have impacted immigrant, refugee, and Indigenous families, who could have faced difficulties in understanding or navigating the application process. Low oral health literacy, particularly among vulnerable populations, could also have reduced the benefit's effectiveness, as some families may not fully recognize the importance of preventive dental care or regular visits to the dentist.

Another key limitation is the absence of data on the actual oral health care received by children. While parents were required to provide proof of at least one dental visit, there were no reporting requirements on the types of care purchased or whether children accessed care beyond an initial exam. This is significant because receiving financial assistance does not guarantee that all of a child's dental needs were met, especially considering the potential inadequacy of $650 for more extensive dental procedures. Furthermore, other social determinants of health, such as transportation barriers, work and time constraints, and challenges related to scheduling appointments, were not addressed by the program and could have further limited access to care.

Finally, there is the potential that some families may have used the funds for other essential needs, as there were no mechanisms to ensure that the benefit was spent solely on dental care. Although parents were required to provide the date of a dental visit, the flexibility of the program might have led to funds being diverted for other purposes, particularly in financially strained households. The new CDCP, functioning as an insurance program, will allow for more detailed data collection, including billing claims, which will facilitate more comprehensive evaluations in the future (12). Overall, this evaluation of the Interim CDB highlight several important areas for improvement, aligning with research recommendations from the recently released Canadian National Oral Health Research Strategy 2024–2030 (22). Future studies and policy refinements should focus on addressing these broader barriers to ensure equitable access to oral healthcare for all children in Canada. The next directions with the CDCP include increasing program engagement with younger age groups and provinces and territories with low child participation rates. Furthermore, dental offices should continue to encourage low-income families to apply to the CDCP now that the Interim CDB ended June 30, 2024. To effectively improve the oral health-related quality of life of Canadian children, a comprehensive approach must be implemented to address unmet social health determinants.

## Data Availability

The original contributions presented in the study are included in the article/Supplementary Material, further inquiries can be directed to the corresponding author.
